# Cement-Based Materials Containing Graphene Oxide and Polyvinyl Alcohol Fiber: Mechanical Properties, Durability, and Microstructure

**DOI:** 10.3390/nano8090638

**Published:** 2018-08-21

**Authors:** Wenguang Jiang, Xiangguo Li, Yang Lv, Mingkai Zhou, Zhuolin Liu, Zhaofeng Ren, Zhuqing Yu

**Affiliations:** 1 State Key Laboratory of Silicate Materials for Architectures, Wuhan University of Technology, Wuhan 430070, China; JWG930329@163.com (W.J.); yang.lv@whut.edu.cn (Y.L.); zhoumingkai@163.com (M.Z.); lin7336@126.com (Z.L.); RZF15671660187@163.com (Z.R.); 2 College of Materials Science and Engineering, Nanjing Tech University, Nanjing 210000, China; zyu@njtech.edu.cn

**Keywords:** graphene oxide, PVA fiber, cement-based materials, mechanical strength, durability, microstructure

## Abstract

The influence of graphene oxide (GO) and polyvinyl alcohol (PVA) fiber on the mechanical performance, durability, and microstructure of cement-based materials was investigated in this study. The results revealed that compared with a control sample, the mechanical strength and durability of cement-based materials were significantly improved by adding PVA fiber and GO. The compressive and flexural strength at 28 d were increased by 30.2% and 39.3%, respectively. The chloride migration coefficient at 28 d was reduced from 7.3 × 10^−12^ m^2^/s to 4.3 × 10^−12^ m^2^/s. Under a sulfate corrosion condition for 135 d, the compressive and flexural strength still showed a 13.9% and 12.3% gain, respectively. Furthermore, from the Mercury Intrusion Porosimetry (MIP) test, with the incorporation of GO, the cumulative porosity decreased from more than 0.13 cm^3^/g to about 0.03 cm^3^/g, and the proportion of large capillary pores reduced from around 80% to 30% and that of medium capillary pores increased from approximately 20% to 50%. Scanning electron microscope (SEM) images showed a significant amount of hydration products adhering to the surface of PVA fiber in the GO and PVA fiber modified sample. The addition of GO coupling with PVA fiber in cement-based materials could promote hydration of cement, refine the microstructure, and significantly improve mechanical strength and durability.

## 1. Introduction

As the main ingredients of concrete and mortar, cement has been widely used in buildings and structures [1]. However, both the concrete and mortar are poor in the tensile strength, strain capacity, and durability due to their brittle cracking defects and loose structure [2]. With the progress of society, traditional cement-based materials are unable to meet the increasing demands for durability and mechanical properties of buildings and structures. Therefore, it is significant for the use of cement-based materials if these issues can be effectually solved. Recently, high-performance and high-durability cement-based materials have been developed rapidly through the use of modification materials such as fibers and nanomaterials to improve the structural functionality [3,4].

Fibers such as steel fiber, basalt fiber (BF), polypropylene (PP) fiber, and polyvinyl alcohol (PVA) fiber are common modification materials used in cement-based materials. It has been proven that the toughness and crack resistance of cement-based materials can be effectively improved by incorporating fibers [5,6,7,8,9,10,11]. The toughening and reinforcing effect of fibers in cement-based materials is closely related to the bonding properties between the fibers and the cementitious components [5,8,9,10,11]. PVA fiber has been proved to be a good toughening and reinforcing admixture for cement-based materials due to its excellent performance in improving mechanical strength [12]. This is because, on the one hand, PVA fiber possesses a high tensile strength, and on the other hand, its hydrophilic surface interacts with cementitious component and could form a strong bonding combination [13,14,15,16]. However, PVA fiber has not yet been demonstrated to prevent microcracks formation or refine pore structure. It was incapable for significantly improving the durability of cement-based materials. 

With the rise of nanotechnology, the application of nanomaterials in cement-based materials for improving properties has become a new research hotspot in recent years [17]. Nanomaterials can fill the voids in cement-based materials and form tighter interfaces between aggregates and cementitious components, which is beneficial to the improvement of mechanical properties and durability [18,19,20]. Nano silica (NS) with a large surface area can increase the pozzolanic activity by acting as a center for crystallization, resulting in the formation of large idiomorphic crystals of Ca-Si composition, thus leading to denser microstructure with reduced porosity and increased mechanical strength [21,22,23]. Carbon nanotubes (CNTs) in cement-based materials could lower the porosity and improve compressive and tensile strength by more than 30% [24]. Unlike other nanomaterials, graphene oxide (GO), the graphene derivative, has excellent mechanical properties whose Young’s modulus and intrinsic strength are evaluated as high as 1 TPa and 100 GPa, respectively [25]. Moreover, owing to the existence of abundant hydrophilic oxygen-containing functional groups, such as hydroxyl, carbonyl, and carboxyl, GO could be dispersed evenly in water [26]. There are several articles that have reported the modification effects of GO on cement-based materials. The research [27] of our group showed that, based on the isothermal calorimeter test, GO could accelerate the hydration rate of cement. Li et al. [28] also reported that the hydration rate of cement was observed to increase with the increase of GO content. Pan et al. [29] showed that with the incorporation of GO, the compressive strength and flexural strength of cement paste increased by 15–33% and 41–59%, respectively. Lv et al. [30] revealed that with the addition of 0.03% GO, the tensile strength, flexural strength, and compressive strength of cement composites increased by 78.6%, 60.7%, and 38.9%, respectively. This can be explained by the function groups of GO providing attachment sites for water and cement, thus acting as the crystal nucleuses for cement hydrates and promoting the hydration rate [31]. The durability of cement-based materials could also be improved by GO introduction. Mohammed et al. [32] demonstrated that the pore structure of cement-based materials could be refined by GO, as a result, the durability increased significantly, and the experimental results also revealed that the cement matrix exhibited high freeze-thaw resistance in the case of GO content.

In summary, PVA fiber has a unique effect on the toughness of cement based materials while GO is beneficial to the improvement of the mechanical strength and durability. Thus, a substantial improvement in comprehensive performance of cement-based materials is expected by utilizing the synergy effect of PVA fiber and GO. The purpose of this study is to investigate the mechanical performances, chloride migration, sulfate corrosion resistance, and drying shrinkage property of cement-based materials modified by GO or/and PVA fiber. Moreover, the synergy effect of GO and PVA fiber on the modified cement-based materials was discussed by means of a pore structure test and scanning electron microscope (SEM) images analysis.

## 2. Experiment

### 2.1. Materials

**Figure 1:**
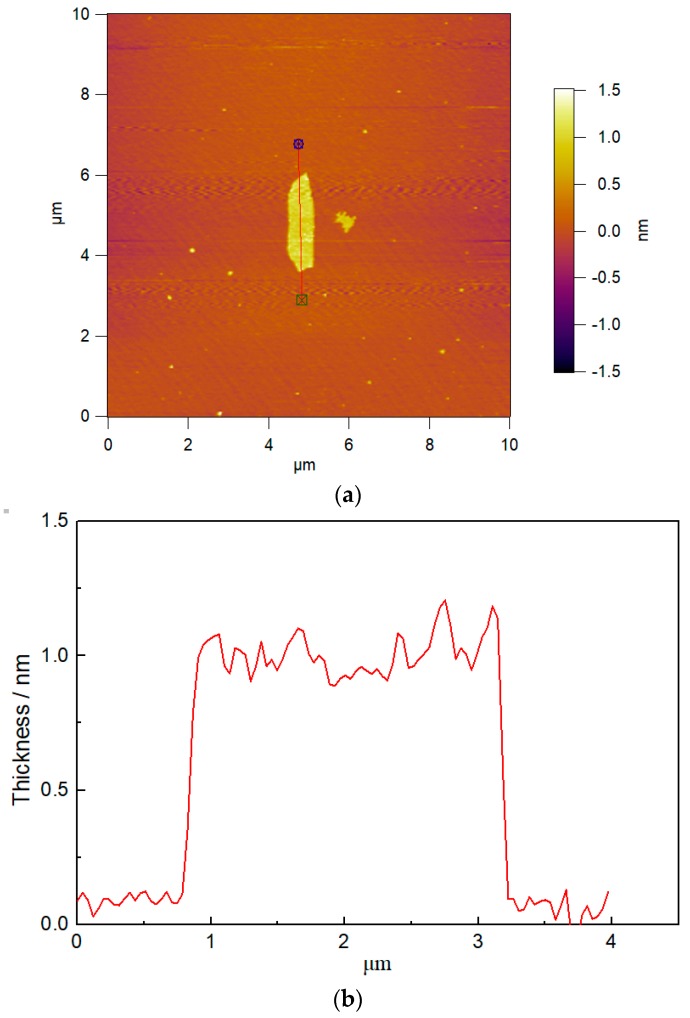
Atomic force microscope (AFM) image of the graphene oxide (GO) (**a**) The length and width of GO; (**b**) The thickness of GO.

GO was prepared in the laboratory, the preparation process and characteristics were described in the previous work [33]. The Fourier-transform infrared spectroscopic (FT-IR) spectrum (Thermo Nicolet, Madison, WI, USA) confirmed that the oxygen functional groups of –OH, –COOH, and –O– were found on the surface of GO. The X-ray diffraction (XRD) spectra (RIGAKU Corporation, Tokyo, Japan) showed the diffraction peak of GO was at 2θ = 9.71°, and the corresponding interlayer spacing was 0.93 nm. In addition, an atomic force microscope (AFM) image (Asylum Research, Santa Barbara, CA, USA) of GO is displayed in Figure 1. It can be seen from the image that the size of the GO sheet reached the nanometer scale, and the thickness of a single irregular sheet was about 1 nm, with its length and width at about 1 µm and 2.5 µm, respectively.

Ordinary Portland Cement (OPC) CEM I 42.5, fly ash (FA), and silica fume (SF) used in the present study were supplied by China Resources Cement Holdings Limited (Wuhan, China), Huaneng Yangluo Power Plant (Wuhan, China) and Wuhan Sentai metallurgy Co. Ltd. (Wuhan, China), respectively. Table 1 presented the chemical compositions of the materials obtained by X-Ray Fluorescence (XRF) (PANalytical B.V., Almelo, The Netherlands) test.

The siliceous sand used in this study was supplied by Xiamen China ISO Standard Sand Co. Ltd. (Xiamen, China) PVA fiber with 12 mm length was obtained from Wuhan Tianhui fiber material Co. Ltd. (Wuhan, China) Polycarboxylate (PC) superplasticizer with 40% solid content and defoaming agents (DA, tributyl phosphate) were produced by Wuhan Huaxuan High-Tech Co. Ltd. (Wuhan, China). The deionized water was used for all mixtures.

### 2.2. Mixing Procedure and Sample Preparation 

Table 2 showed the mixture design for the mortar samples preparation. The amount of the mixing water was the sum of the deionized water and the water introduced by the PC superplasticizer. The dosage of PC referred to the amount of solids in the PC superplasticizer solution. For example, 6.2 g PC was equivalent to 15.5 g of PC superplasticizer solution and 260 g of mixing water that amounted to 250.7 g of deionized water plus 9.3 g of water introduced by the PC superplasticizer solution. In order to ensure uniform dispersion of GO, ultrasonic preprocessing was necessary. The specific process was as follows: First, the PC superplasticizer solution and DA were diluted into 75 wt % deionized water. Then, the weighed GO was added into the mixture solution and gently stirred with a glass rod. After that, the mixture solution was oscillated for 20 min using an ultrasonic machine (SB-5200D) (Ningbo Scientz Biotechnology Co., Ltd., Ningbo, China) with 250 W power and 40 KHz frequency. Besides, the PVA fiber was mixed with sand and binder in a mortar mixer (JJ-5) (Wuxi Jianyi Experiment Instrument Co. Ltd., Wuxi, China) for 2 min at low speed (the rotation rate: 140 ± 5 r/min; the revolution rate: 62 ± 5 r/min). Then, the sonicated GO dispersion was added and the remnant was washed in by the remaining 25 wt % deionized water. Afterwards, the mixture was continually stirred for 1 min at low speed and 2 min at high speed (the rotation rate: 285 ± 10 r/min; the revolution rate: 125 ± 10 r/min). The experiment was carried out at 20 °C.

### 2.3. Characterization of the Cement-Based Materials

#### 2.3.1. Mechanical Performance

The mixtures were casted into 40 × 40 × 160 mm^3^ steel molds twice and vibrated on a vibrostand 60 times for densification after each casting. Then, the surface of the castings were smoothed with a scraper and covered with preservative film. Afterwards, they were placed for 24 h at a temperature of 20 ± 5 °C. After demolding, the specimens were cured under the standard curing condition at constant temperature and relative humidity (RH) (20 ± 2 °C and ≥95% RH) until test age. The mechanical strength test was performed referring to Chinese standard GB/T 50081-2002 at the age of 3, 7, and 28 d [34]. Three samples were used for the flexural strength measurement and six samples left from the flexural test were used for the compressive strength measurement. The loading rates for flexural and compressive strength measurement were 50 ± 10 N/s and 2400 ± 200 N/s, respectively. The mean value of three test results and six test results was obtained for the flexural and compressive strength of each mixture, respectively.

#### 2.3.2. Rapid Chloride Migration Test (RCM)

The mixtures were casted into Φ 100 × 50 mm^2^ plastic molds. The molding process and curing conditions were the same as that of the specimens for mechanical strength measurement. Curing for 28 d, three samples for each mixture were used for the RCM test which was conducted in accordance with Chinese standard GB/T 50082-2009 [35]. In the preparatory phase, the specimens were placed in the vacuum container and the pressure within 1~5 KPa was maintained for 3 h. Saturated calcium hydroxide solution was added to immerse the specimens, and the vacuum level was maintained for one additional hour. Specimens were soaked in the added solution for 18 ± 2 h after turning off the pump. In the test setup, the lateral surface of each specimen was sealed with a rubber sleeve and fixed by two stainless steel hoops (height 25 mm). Then, 0.3 mol/L NaOH solution and 10% NaCl solution were poured into the anode chamber and cathode chamber, respectively. The voltage was 30 ± 0.2 V to record the initial current. Afterwards, the test voltage was adjusted and the test duration was set according to the initial current. Record the initial and final temperatures of anode solution and the final current. After the migration test, each sample was split into two halves and 0.1 mol/L AgNO_3_ solution was sprayed on the fracture surface to confirm the penetration depth of chloride ion.

#### 2.3.3. Sulfate Corrosion Resistant

After standard curing for 28 d, the mortar specimens with a size of 40 × 40 × 160 mm^3^ were immersed in a 5% Na_2_SO_4_ solution. After immersion for 45, 90, and 135 d, compressive and flexural strength measurements of three samples for each mixture were in accordance with the testing procedures in Section 2.3.1.

#### 2.3.4. Drying Shrinkage

The mixtures were casted into 25 × 25 × 280 mm^3^ steel molds (both ends were embedded with gage stud bold) twice, scraped with a scraper, and mashed with a square tamper (23 × 23 mm^2^) 24 times for densification after each casting. After curing in the mold (covered with preservative film) for 24 h at a temperature of 20 ± 5 °C, the specimens were demolded and stored in the room with constant temperature and relative humidity of 20 ± 3 °C and 50 ± 4%. The length of the specimens was recorded immediately after demolding by a comparator (precision: 0.01 mm) and was regarded as the initial length for the drying shrinkage calculation. The drying shrinkage of the specimens was measured up to 90 days after demolding. Three specimens were used for each mixture.

#### 2.3.5. Mercury Intrusion Porosimetry (MIP) analysis

The pore structure characteristics of the cement-based materials at 28 d age were studied by MIP tests which were carried out on a Quanta Chrome Pore Master GT60 mercury intrusion porosimeter (Quanta Chrome, Boynton Beach, FL, USA). For the measurement, the specimens were broken into lumps with diameters ranging from 3 to 6 mm. Afterwards, the lumps were soaked in anhydrous ethanol for more than 24 h to replace the pore water, and then dried at 40 °C for more than 12 h in a vacuum drying oven. During MIP, the high pressure was within the range of 140–420 KPa and the low pressure was within the range of 1.5–350 KPa. Pore size ranging from 0.0035 µm to 200 µm can be recorded.

#### 2.3.6. Microstructure Characterization

Microstructure characteristics of the fracture surfaces of the cement-based materials were studied by scanning electron microscope (SEM) using a high resolution scanning electron microscopy (QUANTA 200 FEG, Field Electron and Ion Company (FEI), Hillsboro, OR, USA) equipped with a cold field emission electron gun operating at 15 kV. For the measurement, the specimens which were kept in anhydrous ethanol after being crushed at 28 d were dried at 40 °C for more than 12 h in a vacuum drying oven. Then, they were processed into lumps with a size of approximately 5 × 5 × 2 mm^3^. The fracture surface was sputter-coated with a thin layer (1 nm) of Pt prior to SEM observation.

## 3. Results and Discussion 

### 3.1. Mechanical Performances

**Figure 2:**
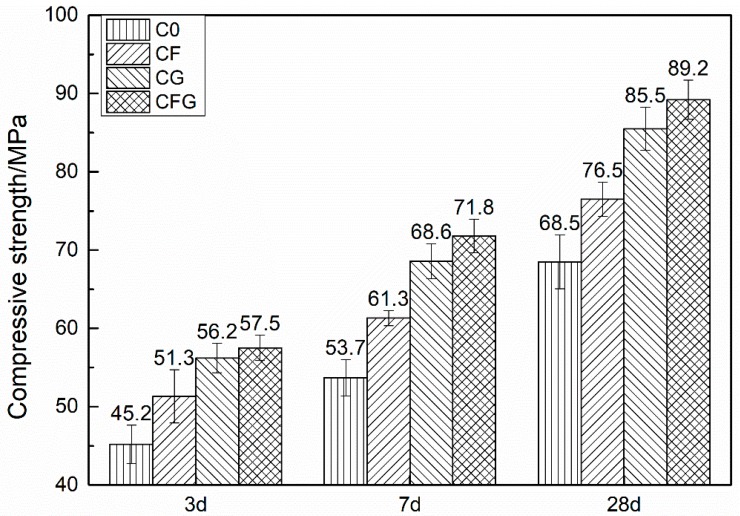
Compressive strength vs. curing time.

**Figure 3:**
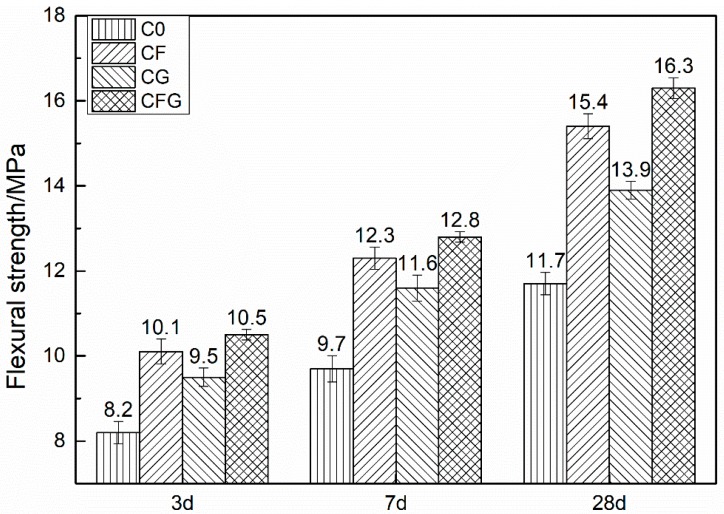
Flexural strength vs. curing time.

The compressive and flexural strength of cement-based materials modified by GO and/or PVA fiber are summarized in Figure 2 and Figure 3, respectively. C0 represented the control sample, CF and CG represented the PVA fiber and GO modified sample, respectively. CFG represented the GO coupling with PVA fiber modified sample.

It is obvious from the figures that the addition of PVA fiber and/or GO could increase both the compressive and flexural strength of the mixtures. The incorporation of PVA fiber into the mixtures lead to an increased compressive strength with an improvement of more than 10% at each age, and the flexural strength was increased by 23.2% and 31.6% at 3 d and 28 d, respectively. It can be concluded that PVA fiber has a substantially more pronounced effect on the flexural strength than the effect on the compressive strength. According to the previous studies [36,37], PVA fiber can act as a bridge in cement-based materials, which not only inhibits the expansion of the crack but also consumes fracture energy in the process of being pulled out or tensile failure, leading to mechanical strength improvement, particularly the flexural strength. Differently, with the addition of GO into cement-based materials, the increment in compressive strength was more prominent. At GO content, the value of the increase rate for the compressive strength improvement was approximately 25% whereas for flexural strength improvement it was less than 20% at each age. As previously stated by Wang et al. [38], GO was involved in the cement hydration, accelerating the nucleation, growth, and phase separation process of hydration products, which promotes the hydration rate and improves the crystal order, thus enhancing the mechanical strength of the cement-based materials. When the cement-based materials were modified by PVA fiber coupling with GO, the compressive strength was increased by around 30% at each age. The value of the increase rate for the flexural strength improvement was from 28% to 39.3% with the curing age increasing from 3 d to 28 d. The compressive strength was mainly increased by GO, whereas the flexural strength was increased both by GO and PVA fiber. 

### 3.2. Impermeability

**Figure 4:**
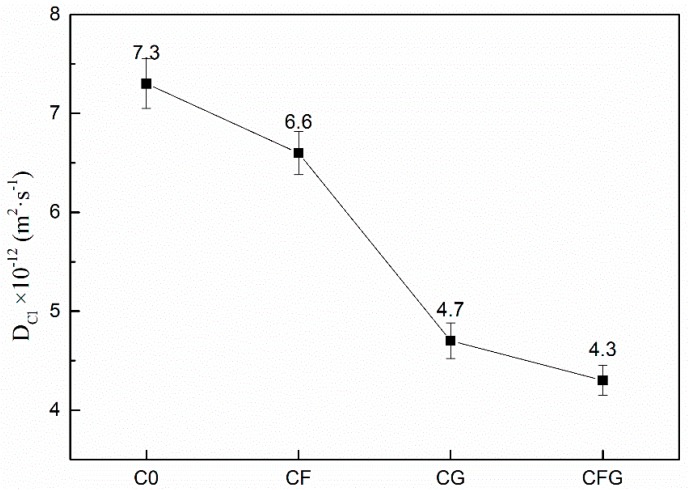
Chloride migration coefficient of samples.

Chloride migration coefficient is one of the important indexes to characterize the resistance to chloride ion permeation of cement-based materials. The chloride migration coefficient of C0, CF, CG, and CFG are shown in Figure 4.

As shown in Figure 4, the chloride migration coefficient of the mixtures was decreased due to the incorporation of PVA fiber and/or GO. Therein, the effect of PVA fiber was minimal, which just led to the chloride migration coefficient decreasing from 7.3 × 10^−12^ m^2^/s for C0 to 6.6 × 10^−12^ m^2^/s for CF, the decrease magnitude was less than 10%. Moreover, the effect of GO was significant; compared with C0, the chloride migration coefficient of CG was decreased by 35.6%. Furthermore, the coupling effect of PVA fiber and GO resulted in a further decrease in the chloride migration coefficient of CFG, and the decrease magnitude reached 41.1%.

A previous study [39] revealed that cracks may become paths for chloride ions to penetrate into cement-based materials. PVA fibers mainly act as bridges in the cement matrix, thus restraining the expansion of cracks [36,37], which blocked the paths for chloride ion penetration. As a result, the chloride migration coefficient of CF was slightly decreased. Besides, as stated in previous literature [40,41,42,43], chloride ion penetration was closely related to the pore structure of cement-based materials. The chloride migration coefficient showed a linear increase with the pore volume and critical pore diameter. GO in cement-based materials can fill in the micropores and refine the pore structure. Moreover, the nucleation seed effect of it during cement hydration could accelerate the hydration reaction rate, promoting the accumulation of hydration products, thus leading to a denser microstructure with decreased porosity [44]. This explained the reason of the significant decrease in the chloride migration coefficient of CG. As for CFG, on the one hand, PVA fiber inhibited the expansion of the crack, on the other hand, GO refined the microstructure, both actions blocked the path for chloride ion penetration. As a result, the chloride migration coefficient was minimal.

### 3.3. Sulfate Corrosion Resistance

**Figure 5:**
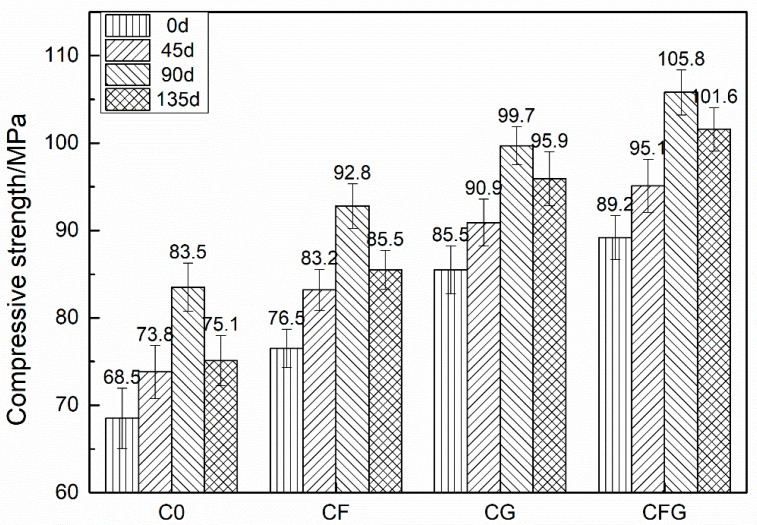
Compressive strength of samples under the sulfate corrosion condition.

**Figure 6:**
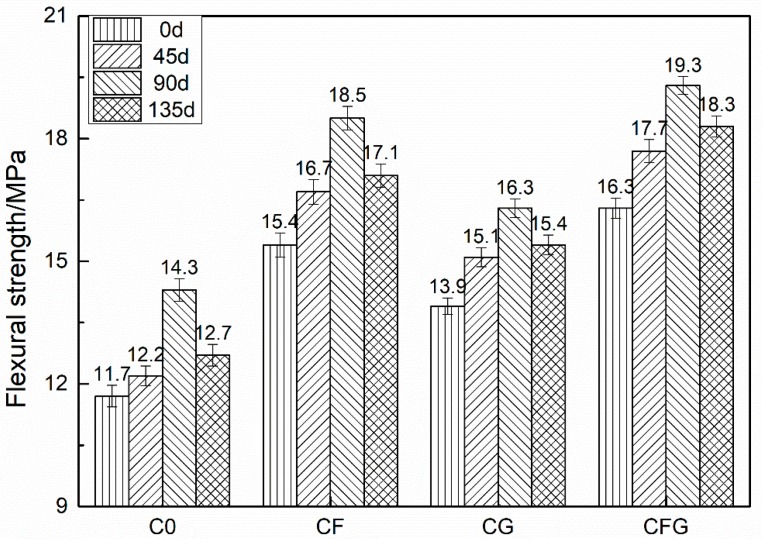
Flexural strength of samples under the sulfate corrosion condition.

The compressive and flexural strength of C0, CF, CG, and CFG after being immersed in a 5% Na_2_SO_4_ solution for different times are shown in Figure 5 and Figure 6, respectively.

As shown in the figures, the mechanical strength of specimens showed a continuous increase with the soaking time increasing to 90 d. Afterwards, the specimens showed a decreased mechanical strength with the soaking time increasing to 135 d. The mechanical strength of specimens soaked for 135 d still showed higher mechanical strength compared with that of the specimens before sulfate solution soaking. The values of the compressive strength rates of C0, CF, CG, and CFG were increased from approximately 7% at 45 d to 20% at 90 d and then decreased to around 11% at 135 d. The value of the flexural strength increase rate showed a similar trend.

Under sulfate corrosion conditions, the mechanical strength of mortar was mainly affected by two main factors. The positive factor related to the continuous hydration of unhydrated cement in the specimens after 28 d standard curing, which can produce more hydrates and lead to a more compact mortar matrix with improved mechanical strength. The negative factor was due to the sulfate corrosion process. The SO_4_^2−^ ions penetrated into mortar and reacted with the components of the cementitious materials to form intumescent minerals, such as ettringite, resulting in expansion stress and cracks in the mortar with decreased mechanical strength. Both the positive effect due to the continuous hydration and negative effect regarding the sulfate corrosion acted simultaneously. However, at the early stage of sulfate corrosion, the positive factor was dominant, therefore, the mechanical strength of mortar was increased. With the increase of corrosion time, the negative factor became more and more prominent, thus, the mechanical strength begun to decrease. 

In addition, as GO could accelerate the hydration reaction rate of cement [27,28,44], the amount of unhydrated cement in the mixture CG or CFG was limited. Consequently, at the early stage, the dominant positive effect due to the continuous hydrations was limited, leading to a minor mechanical strength increase of CG and CFG at 90 d. At the stage of mechanical strength degradation, PVA fibers could counteract the expansion stress and restrain cracks propagation caused by the intumescent minerals. Meanwhile, similar to the case of improved resistance to chloride ion permeability, GO could also improve the resistance to SO_4_^2−^ permeability of the mixture, which prevented the formation of intumescent minerals in the mortar. Therefore, due to the coupling action of PVA fiber and GO, the mechanical strength of CFG was maintained at a maximum among the four series of specimens at 135 d.

### 3.4. Drying Shrinkage

**Figure 7:**
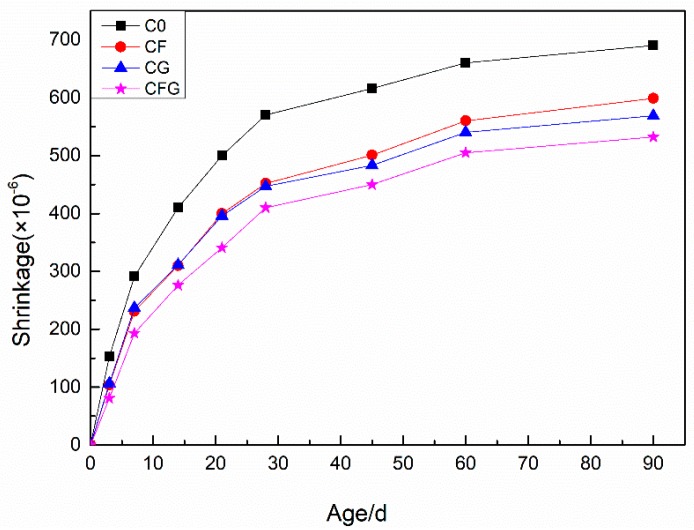
Drying shrinkage rate of specimens at different ages.

Figure 7 shows the shrinkage-time relationship of specimens which developed over a period of 90 d of air storage. All specimens with the addition of PVA fiber and/or GO were found to exhibit a lower total drying shrinkage than the control sample between 0 to 90 d. With PVA fiber content, the 90 d shrinkage of CF was decreased by 13.2% compared with C0. Moreover, with the incorporation of GO, the 90 d shrinkage of CG decreased by 17.5%. Furthermore, when adding PVA fiber coupling with GO, the drying shrinkage of CFG was minimal at all ages and the 90 d shrinkage was decreased by 22.9%.

The main reason for drying shrinkage was the free water evaporation from the capillary pores through the surface of the cement-based materials, which was exposed to a low RH environment. Capillary forces would hold the free water in the capillary pores. In addition, as a function of pore size diameter, the smaller the pore diameter, the more powerful is the capillary force [45]. In other words, when the pore structure of cement-based materials was more refined, the evaporation of free water is slower, and, thus, the drying shrinkage is smaller. In addition, improving mechanical properties of cement-based materials was also beneficial in the enhancement its ability to resist shrinkage. As discussed before, GO in the cement matrix, on the one hand, filled in the micropores and refined the micropore structure, on the other hand, acted as a nucleation seed to accelerate the hydration reaction rate of cement, thus leading to a denser microstructure, improved mechanical strength [44], and decreased drying shrinkage of mixtures containing GO. Besides, Zhang and Li [46] revealed the possible mechanism for the positive effects of fibers on drying shrinkage: when shrinkage caused tensile stresses in the cement matrix, fibers restrained shrinkage by shear along the fiber-matrix interface.

### 3.5. Pore Structure Analysis

**Figure 8:**
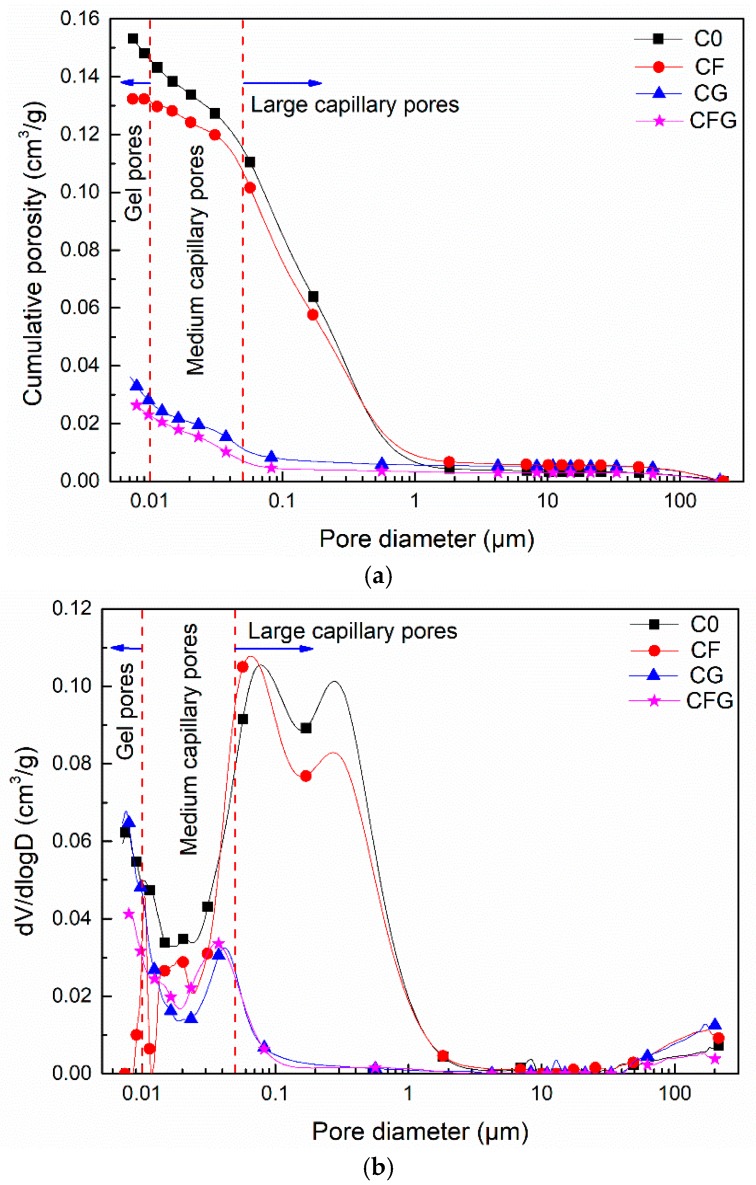
(**a**) Cumulative porosity; and (**b**) Log-differential volume curve plots.

The pore structure of C0, CF, CG, and CFG at 28 d was determined by means of Mercury Intrusion Porosimetry (MIP). The porosity and pore size distribution results are summarized in Table 3, and their curves are shown in Figure 8a,b. According to previous research, the pores can be divided into three size domains: >0.05 μm, 0.05–0.01 μm, and <0.01 μm, which correspond to large capillary pores, medium capillary pores, and gel pores, respectively [47].

As shown in Table 3 and Figure 8a, the cumulative porosity of C0, CF, CG, and CFG were 15.43 × 10^−2^ cm^3^/g, 13.23 × 10^−2^ cm^3^/g, 3.62 × 10^−2^ cm^3^/g and 2.67 × 10^−2^ cm^3^/g, respectively. Compared with C0, the cumulative porosity of CF, CG, and CFG was decreased by 14.26%, 76.54%, and 82.70%, respectively. Figure 8b shows a different pore size distribution for the cement-based materials. The pore structure of C0 and CF are dominated by large capillary pores, while that of CG and CFG were mainly medium capillary pores. According to Table 3, the proportion of large capillary pores of C0 and CF were 74.40% and 80.88%, respectively, which decreased to 30.66% for CG and to 25.84% for CFG, meanwhile, the proportion of medium capillary pores was increased from 11.48% for C0 and 10.7% for CF to 45.58% for CG and to 59.55% for CFG. In addition, the total intrusion (cm^3^/g) of gel pores, usually belonging to C–S–H channels, should increase with the addition of GO, since GO could promote the formation of hydration products. However, the results showed no difference in the gel pore volume between the control specimen and the GO modified specimen. This may be because GO could regulate the crystallization of hydration products [38] and the C–S–H grows within the inter-hydrate spaces (around 8–10 nm), leading to a transition from the growth of “less dense” to “more dense” products [48]. The transition resulted in decreased gel porosity.

The above results indicate that the addition of PVA fiber in cement-based materials only showed a slight effect on pore structure, but GO incorporation can significantly decrease the porosity and refine the pore size. It also proved the statement discussed in the previous section that GO in cement-based materials could not only fill in the micropores but also act as crystallization centers for hydrated products to accelerate the hydration reaction rate, leading to a refined microstructure.

### 3.6. SEM Observations

**Figure 9:**
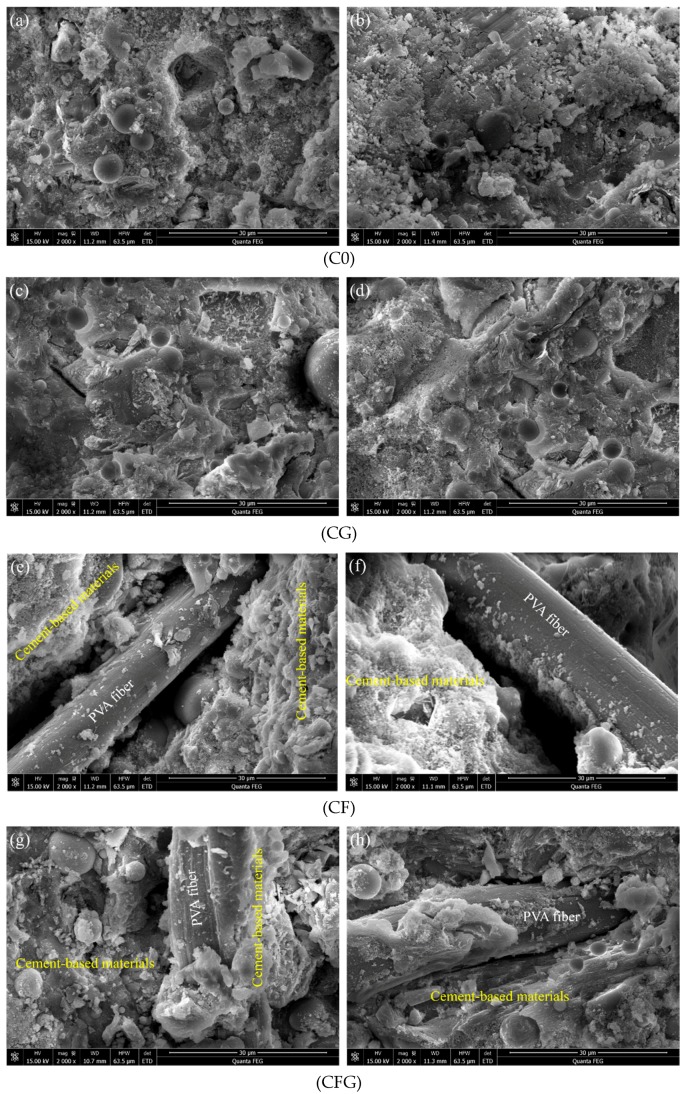
Microstructure morphology of hardened specimens at 28 d age at 2000 magnification; (**a**,**b**) control sample; (**c**,**d**) GO modified sample; (**e**,**f**) PVA fiber modified sample; (**g**,**h**) GO coupling with PVA fiber modified sample.

Microstructure morphology of hardened specimens at 28 d was evaluated by means of SEM. Two images were captured of each specimen at different positions. The results are shown in Figure 9.

As shown in Figure 9a,b, there was loose-pellet-like C–S–H gel crystal nucleuses and unhydrated FA particles in the microstructure of C0. Comparatively, Figure 9c,d show a compact and uniform microstructure of CG. The observation indicated that with the addition of GO, the microstructure of the cement matrix was refined, which can be due to GO being involved in the cement hydration, accelerating the nucleation, growth, and phase separation process of hydrated products, thereby promoting the hydration rate and improving the crystal order [38]. Moreover, differences between Figure 9e,f and Figure 9g,h can be observed by comparing carefully. Figure 9g,h show not only a compact microstructure of cement matrix but also abundant hydration products adhering to the PVA fiber surface. These micrographs reveal that GO in PVA fiber modified cement-based materials could promote the formation of hydration products to densify the microstructure and improve the adhesion of hydration products on PVA fiber, which are beneficial to the combination between PVA fiber and the cement matrix.

## 4. Conclusions

In this study, the influence on the properties of mortar modified by PVA fibers and GO, including mechanical properties, durability and pore structure, were investigated. Based on the results obtained, the following conclusions can be drawn. 

PVA fiber in cement-based materials can effectively improve their toughness and ability of resist crack, thus resulting in an evident improvement of mechanical strength. The inclusion of GO in cement systems was beneficial for pore modification, which in turn enhanced the mechanical and durability properties of the cement-based materials. Moreover, the mechanical and durability properties of cement-based materials can be further improved by the coupling modification of PVA fiber and GO. With the addition of both PVA fiber and GO, the compressive and flexural strength of mortar at 28 d was increased by 30.2% and 39.3%, respectively; meanwhile, the durability properties, including chloride ion impermeability, sulfate corrosion resistance, and drying shrinkage property, were clearly improved.

It can be found from the MIP analysis that the addition of GO in cement-based materials could lead to a decreased cumulative porosity, the value of the decrease rate was about 80%, compared with C0. Furthermore, the addition of GO can refine the pore structure, which means the proportion of large capillary pores reduced from around 80% to 30% and the proportion of medium capillary pores and gel pores, respectively, increased from approximately 20% and 5% to 50% and 20% with the incorporation of GO.

The SEM images showed that the addition of GO in cement-based materials made the microstructure more compact and uniform, meanwhile, it improved the adhesion of hydration products on the PVA fiber, which was beneficial to the combination between PVA fiber and the cement matrix. The mechanical strength of the PVA fiber coupling with the GO modified specimen was higher than that of the PVA fiber without the GO modified specimen.

Since the cement-based materials modified by GO coupling with PVA fiber exhibited excellent mechanical strength and durability, it can be widely used in buildings and structures, such as roads, bridges, dams, etc.

## Figures and Tables

**Table 1:** Chemical composition of ordinary Portland cement (OPC), fly ash (FA), and silica fume (SF).

Compositions	OPC	FA	SF
SiO_2_	21.99	51.53	89.63
Al_2_O_3_	5.92	27.78	0.12
Fe_2_O_3_	3.26	4.73	0.35
CaO	58.64	5.21	3.21
MgO	1.98	1.03	2.82
K_2_O	0.74	1.13	1.01
Na_2_O	0.27	0.62	0.42
SO_3_	2.6	1.87	0.92
Loss on ignition (LOI)	3.5	3.16	1.16

**Table 2:** Mix proportion of cement-based materials. C0 represents the control sample, CF and CG represent the polyvinyl alcohol (PVA) fiber and GO modified sample, respectively. CFG represents the GO coupling with the PVA fiber modified sample. PC = polycarboxylate; DA = defoaming agents.

Samples	OPC/g	FA/g	SF/g	Sand/g	Water/g	GO/g	PVA/g	PC/g	DA/g
C0	620	300	80	1000	260	0	0	6.2	0.5
CF	620	300	80	1000	260	0	5	6.2	0.5
CG	620	300	80	1000	260	0.5	0	6.2	0.5
CFG	620	300	80	1000	260	0.5	5	6.2	0.5

**Table 3:** Porosity and pore size distribution of samples.

Samples	Porosity	Pore Size					
		<0.01 μm		0.01~0.05 μm		>0.05 μm	
	10^−2^ cm^3^/g	10^−2^ cm^3^/g	%	10^−2^ cm^3^/g	%	10^−2^ cm^3^/g	%
C0	15.43	0.85	5.51	3.10	20.09	11.48	74.40
CF	13.23	0.08	0.60	2.45	18.52	10.7	80.88
CG	3.62	0.86	23.76	1.65	45.58	1.11	30.66
CFG	2.67	0.39	14.61	1.59	59.55	0.69	25.84
